# Navigating PINK1 Parkinson’s Disease: Narratives of Pacific Patients and Caregivers

**DOI:** 10.1155/padi/4024943

**Published:** 2026-09-24

**Authors:** Fuafiva Fa’alau, Atefeh Kiadarbandsari, Miraneta Foroti, Richard Roxburgh, Christina M. Buchanan

**Affiliations:** ^1^ Department of Pacific Health, Faculty of Medical and Health Science, The University of Auckland, Private Bag, 92019, Auckland, New Zealand, auckland.ac.nz; ^2^ Neurology Department, Auckland City Hospital, Te Whatu Ora–Health New Zealand, Private Bag, 92024, Auckland, New Zealand, adhb.govt.nz; ^3^ Centre for Brain Research, Faculty of Medical and Health Science, The University of Auckland, Private Bag, 92019, Auckland, New Zealand, auckland.ac.nz

## Abstract

Parkinson’s disease in its common form is a sporadic disease of older age affecting men more than women, with onset after the age of 60 years. However, Parkinson’s disease, caused by variants in the PINK1 gene, affects males and females equally and causes early‐onset Parkinson’s disease. Furthermore, although it is rare elsewhere in the world, it is relatively common among Pacific populations, where carrier rates can reach 1 in 18 for a specific variant. All these differences between PINK1 Parkinson’s disease and typical Parkinson’s disease would be expected to affect people differently; however, the understanding of how this form of Parkinson’s disease affects Pacific peoples is limited. Exploring the lived experiences of people with PINK1 Parkinson’s disease and their caregivers is essential for informing culturally responsive care. We employed a specific Pacific qualitative research approach called Talanoa, which is centred on storytelling and open dialogue sessions, to explore the experiences of Pacific people with PINK1 Parkinson’s disease and their caregivers. Data were analysed thematically. We studied 16 participants (11 patients and 5 caregivers). Two main themes were identified: *Adaptation Struggles* and *Positive Adaptation*. *Adaptation Struggles* captured personal (e.g., impaired mental state and medication management), social (e.g., progressive disengagement from social and community life), cultural (e.g., stigma and the mismatch between cultural beliefs and biomedical perspectives of Parkinson’s disease) and systemic challenges (e.g., limited awareness and unmet needs). *Positive Adaptation* reflected participants’ efforts towards normalisation and reliance on support networks such as family, church and community. Enhancing services and awareness of PINK1 Parkinson’s disease through culturally responsive care delivered by healthcare providers and health workers can facilitate early detection, improve medical management and enhance psychosocial well‐being. Collaboration among researchers, clinicians and Pacific communities is vital to reducing diagnostic delays and improving long‐term outcomes.

## 1. Introduction

Idiopathic Parkinson’s disease is a sporadic disease of older age affecting men more than women, with onset after the age of 60 [1]. However, Parkinson’s disease caused by variants in the PINK1 gene is a monogenic, autosomal recessive form of early‐onset Parkinson’s disease (EOPD) that may cluster within families and shows no significant sex bias, with onset often occurring before age 40 and including juvenile cases [2–5]. PINK1 Parkinson’s disease can be complex to diagnose, partly because it manifests differently, with a predominance of dystonia early in the disease and partly because of the early onset, as doctors tend not to consider this diagnosis in this age group. This can affect access to medication.

PINK1 Parkinson’s disease is extremely rare in the world, accounting for less than 1% of Parkinson’s disease [6–8]. However, the presence of a specific variant (PINK1 p.L347P) in Pacific people [9–11] where carrier rates are as high as 1 in 16 for a specific variant [10] means that the condition is common in select populations—for example, projected to be near one in a thousand people in Samoa—and Pacific people have their own unique social, cultural and emotional experiences of the world, health and medicine. All these factors make it impossible to generalise from the bulk of research on idiopathic Parkinson’s disease conducted in Western and European populations [12].

About half of people with Parkinson’s disease experience social withdrawal or social phobia, resulting in less contact with family and friends, being more homebound and facing reduced workforce participation [13]. This is particularly the case for patients with EOPD who may have symptoms in the prime of their working life or even before they have finished their education, leading not only to reduced income and financial insecurity [14] but also to effects on their self‐image with respect to establishing a career and providing for their family. In addition, caregivers are also affected–frequently experiencing stress, depression and burnout, withdrawal from work and social life, particularly as the disease progresses [15, 16].

This study was undertaken primarily in Auckland, which is home to one of the largest Pacific (Polynesian) populations in the world, with Pacific peoples comprising approximately 16.6% of the city’s population in the 2023 New Zealand census [17]. For Pacific people in New Zealand, reduced access to healthcare and preventive services (e.g., primary care) contributes to health disparities, as such limitations directly affect health outcomes and quality of care [18, 19]. In this sense, the challenges associated with Parkinson’s disease—including difficulties in early diagnosis, complex symptom management and the absence of disease‐modifying therapies [1]—may have a substantial impact on Pacific patients and their caregivers. Exploring the lived experiences of Pacific people with PINK1 Parkinson’s disease and their caregivers can help illuminate the cultural, social and emotional dimensions of living with a genetically driven neurological condition. This study aims to capture these narratives and understand how individuals and families navigate the illness and care journey.

## 2. Methods

This study employed a Talanoa research approach, a Pacific research methodology centred on storytelling, open dialogue and relational trust. It prioritises participant‐led, empathetic conversations that allow people to share their experiences in a culturally safe and nonhierarchical way, making it especially useful for qualitative research in Pacific communities [20–22].

### 2.1. Participants

The study included 16 participants, including 11 patients and five caregivers. Four participants lived outside Auckland, in Waikato, Christchurch and Invercargill. The rest of the participants were based in Auckland. Participants received information about study procedures and provided written informed consent before participating. Ethics approval was granted by the New Zealand Health and Disability Ethics Committee (HDEC 2022 EXP11739). Table 1 provides an overview of participants’ demographic characteristics and the method of data acquisition. Participants are identified by type and number: ‘P’ for patients and ‘C’ for caregivers, followed by a unique participant number (e.g., P1 refers to Patient 1).

**TABLE 1:** Participant demographic and clinical characteristics and data acquisition procedures.

Patient	Gender	Age range	Ethnicity	Age of onset	Caregiver	Data acquisition procedure
P1	Male	55–59	Samoan	39	C1 was the wife and caregiver of P1	Group Talanoa with FF, MF, CB
P2	Male	55–59	Tongan	36	N/A	Individual Talanoa with FF and MF
P3	Male	50–54	Tongan	33	N/A	Individual Talanoa with MF
P4	Female	30–34	Samoan	23	C2 was the father and caregiver of P4	Individual Talanoa with FF and MF
P5	Female	55–59	Tokelauan	45	N/A	Individual Talanoa with MF
P6	Male	45–49	Tokelauan	34	C3 was the wife and caregiver of P6	Individual Talanoa with MF by phone
P7	Male	55–59	Tongan	24	C4 was the wife and caregiver of P7	Group Talanoa with FF, MF, CB
P8	Male	65–69	Samoan	29	C5 was the wife and caregiver of P8	Group Talanoa with FF, MF, CB
P9	Female	50–54	Samoan	40	N/A	Individual Talanoa with CB
P10	Female	25–29	Samoan	22	N/A	Individual Talanoa with CB via Zoom
P11	Male	45–49	Samoan	43	N/A	Individual Talanoa with CB

### 2.2. Procedure

Recruitment initially focused on the South Auckland Pacific Parkinson’s Support Group. Initial participation took place in a Talanoa focus group discussion, consisting of P1, C1, P7, C4 and C5, together with researchers FF, MF and CB. Individuals who were unable to attend the focus group Talanoa were subsequently invited to take part in individual interviews. Talanoas were arranged by members of the research team (MF and CB), who contacted participants directly to schedule interview times if they were willing to participate, either in person, by phone or via Zoom. Patients who wished to include their caregivers in the Talanoa were accommodated, and caregivers were consented before proceeding with the Talanoa. Data were collected from March 2023 to August 2024 by FF, MF and/or CB, and data collection concluded when saturation was reached. FF, CB and MF are all professional and academic researchers who have no formal or direct relationships with the participants. All three conducted the interviews and the focus group for this study. FF and MF are native speakers of Samoan; therefore, portions of the Talanoa for P1, C1, P4, C2 and C5 were conducted in Samoan.

### 2.3. Analysis

The Talanoas were transcribed by MF and CB. Data were analysed using Braun and Clarke’s [23] six‐phase reflexive thematic analysis, which is particularly appropriate for exploring underresearched areas by FF, MF and AK. The stages include familiarising with the data, generating initial codes, searching for themes, reviewing themes, defining and naming themes and producing the report. Initially, the first coder familiarised herself with the data through repeated readings and noted preliminary ideas. Using an inductive coding approach, meaningful data segments were identified and coded, and related codes were organised into broader patterns using NVivo. The first coder was MF, a Pacific researcher who transcribed the data and conducted the first stage of analysis, which involved familiarising with the data and identifying items of potential interest. She noted down initial ideas and then generated initial codes inductively by identifying and classifying meaningful data segments (the second stage). Related codes were then grouped and refined into broader themes representing patterned meanings (the third stage). Six themes were identified at this stage. In the fourth stage, another researcher, AK, with greater experience in six‐phase reflexive thematic analysis, engaged in the coding. During the reviewing phase, the second coder collaborated to refine and validate the emerging themes, ensuring consistency with the dataset and alignment with the research question. The initially identified themes were conceptually appropriate but required refinement and renaming. For example, the first coder labelled one subtheme as ‘Positive look at life’, whereas the second coder, with a psychology background, recognised that a positive outlook can signify a coping strategy (e.g., positive refocusing) that does not necessarily involve behavioural change. In contrast, our participants did not simply adopt positive thinking; they actively took steps to hold a sense of normality in their daily lives. Consequently, the subtheme was renamed to reflect its underlying meaning better. Furthermore, the themes appeared to form two broader, contrasting clusters. Thus, first‐level themes and related subthemes were clearly defined, named and described to capture their central meanings. Stages four and five of reflexive analysis were conducted iteratively, with the MF, FF and AK moving back and forth between the data and the preceding stages of analysis. The other coauthors, CB and RR, were invited to evaluate the analysis, which helped refine the final stage of the analytic process. For example, one coauthor suggested reorganising the themes and subthemes to produce a clearer, more coherent thematic map in which patients’ struggles are presented, followed by factors that can help them overcome those challenges. Finally, a comprehensive report was developed, supported by illustrative quotations and iteratively revised to ensure accuracy, clarity and analytical rigour.

## 3. Results

Eleven Pacific patients diagnosed with PINK1 Parkinson’s disease, and five caregivers were recruited. Of the 11 patients, eight lived with their families. The remaining three participants lived alone; two maintained regular contact with family members, while one lived without family support. Five patients participated with their family caregivers. There were no dropouts. Our participants reported EOPD, with symptom onset before the age of 50 years.

**FIGURE 1:**
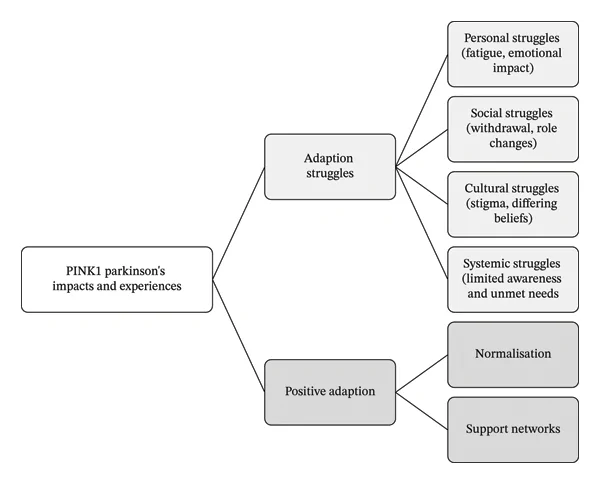
Thematic map of Pasifika PINK1 Parkinson’s patients’ and caregivers’ experiences.

Two key themes emerged from the analysis of Talanoa discussions with Pasifika individuals living with PINK1 Parkinson’s disease and their caregivers, highlighting their daily experiences with the illness. As shown in Figure 1, these themes encompassed two subthemes: *Adaptation Struggles and Positive Adaptation*. *Adaptation Struggles* captured personal, social, cultural and systemic challenges. *Positive Adaptation* was reflected through participants’ experiences of normalisation and support networks. Consistent with participants’ identification codes in Table 1, their quotes are presented by the same type and number: ‘P’ for patients and ‘C’ for caregivers, followed by a unique participant number (e.g., P1 refers to Patient 1) in this section.

### 3.1. Theme of Adaptation Struggles

One theme, termed *Adaptation Struggles*, referred to the personal, social, cultural and systemic challenges participants experienced in adapting to their condition.

#### 3.1.1. Personal Challenges

Participants and caregivers consistently described the profound negative impacts of living with PINK1 Parkinson’s disease, which significantly affected their mental state and required medication management. Discussions frequently centred on feelings of depression, frustration and a lack of acceptance, which contributed to the development of negative thoughts. Reflecting on their experience, a participant stated, *‘I hate it, I really hate having Parkinson’s, but I can’t do anything about it so I just have to live with it’. (P5).* A caregiver also shared, *‘I don’t know how to describe it, but everybody knows that he’s like this nice, happy guy but he really struggles with a little bit of depressive thoughts and stuff’. (C3).* Another caregiver highlighted a similar issue, *‘He locked himself in the room when there are visitors that come into the house and ahh it takes ages for him to come out and accept that he’s got Parkinson’. (C4).* One patient described her situation after diagnosis and shared, *‘I absolutely felt that way* [broken] *for the first few months. I felt like I couldn’t be a mother, I couldn’t be a wife, I couldn’t work for my family or provide for my family’. (P10).*

PINK1 Parkinson’s disease imposes significant mental strain, as individuals must meticulously organise their daily activities around the rigid schedule of medication required to control their symptoms. As one participant explained, *‘So how I plan my day I try and take my medications now blah, blah, blah. So it will take me to this hour so what can I do between that hour and now. This is especially the weekends I try to plan the whole day so my pills will last’. (P6).* His caregiver mentioned the same challenge and noted, *‘So on a work day some days are bad. Some days there are struggles but he tries to plan his medication so that he can do the day at work. But we’ve had to have some conversations around because his work wants him to work different shifts’. (C3).*

#### 3.1.2. Social Challenges

Social struggles refer to participants’ impairment in social functioning. Patients reported feelings of social exclusion and embarrassment due to the impact of the condition on their bodies. Some participants described withdrawing from society, including resigning from their jobs, avoiding social interactions with friends and family and being unable to maintain a normal, regular routine. A patient expressed, *‘I was getting paranoid that people behind me were laughing at me so I kind of gave up on that. I know I could have worked from home but I have just not found a job that can do that… Because it is embarrassing it is like an old person’s disease’. (P5*). Another patient also mentioned embarrassment in their narrative as ‘*You really can’t go and socialise… Yeah pretty embarrassed. Maybe it will be all areas of life’. (P3*). Parkinson’s can impact even those who are typically social, which was evident in the account of one participant, *‘I’m a pretty social person, yeah I can tell friends now I don’t go anywhere. Every now and then I go to half an hour away drives. But yeah, other than that, I don’t hang out anymore’. (P6*).

PINK1 Parkinson’s disease can significantly alter an individual’s life, as reflected in these quotations. One participant describes how, following her diagnosis, she is no longer able to engage in activities she once enjoyed. She stated, *‘It tends to stop things I like to do. I like to play rugby and I like to play any sports. You know being out with friends. Yeah well it stops me from doing things I like to do’. (P4*). The following excerpt highlights the experiences of participants and their caregivers regarding societal negative comments and remarks: *‘He didn’t like to go out in Public because people don’t know, like when there’s someone young like him, they look at him and they think it’s like drugs or something. Right because he’s like shifting and uncomfortable. So I think he’s like I don’t know, he would get a lot of stares and stuff like that’. (C3*). A patient noted that she faced work‐related challenges without medication, *‘Without medication I was still a bit restricted. Um, so even having meetings at work, I wasn’t able to confidently sit there and not tremble’. (P10).*

#### 3.1.3. Cultural Challenges

Cultural struggles arise when Pacific cultural beliefs and values do not align with the nature or progression of Parkinson’s disease. Cultural beliefs influence Pacific people’s perceptions and experiences of PINK1 Parkinson’s disease, as reflected in participants’ engagement with traditional healers, spirituality and community stigma. In this context, participants shared their experiences with traditional healers during the focus group. When a participant experienced a change in his body, he and his family immediately sought assistance from traditional healers in Samoa. In such contexts, it is commonly believed that unusual occurrences are attributed to ancestral curses, stemming from perceived wrongdoing. To address these situations, traditional medicines or rituals are employed to lift the curse. As one participant recounted: *‘I went to F’s place in L and said that this lady the su’e ma’i* [traditional Samoan healer] *told me to go and apologise to F… I said, “Please forgive me, Sir, I came on the su’e ma’i′s instructions to apologise.” But nothing happened, no change at all for the girl. Later, my parents said, “Your story is wrong… you shouldn’t come and cause trouble.” (laughs)’ (P1).* This story illustrates how following traditional advice can intersect with family expectations and community norms, creating situations that participants find simultaneously challenging and amusing. A Tongan caregiver continued, *‘We went through that too, some people say its these things from way back. Assume things yeah and we follow and thought yeah you are going to get better and ahh the massage and they use their elbow* [for massage] *and say see they are getting better. No, no don’t go there anymore’. (C4).*

Traditional healers often provided their own interpretations of participants’ symptoms, which sometimes conflicted with biomedical explanations and caused confusion for both participants and caregivers. As one participant described: *‘O lesi la na faimai ole uaua ua kele ona fofo a’u ile uaua* [One healer said it was the muscle, then I was treated for the muscle]*. Ua amata le fofo and lesi mea* [the treatment started and then] *I feel ua malō ia lo’u ua* [my neck is stiff]*. I think that is another sign you know’. (P1).* And a caregiver added: *‘You know it is great for the physio, see we have a physio here so there you go. I also know there is this thing called dystonia yea which is more to do with cramping of the muscles would that be* [the cause?]. *Because those are the original signs of …* [mentioning the patient in their family], [is] *the cramping i oga vae* [in his legs] *that’s where the most common ones that always come up ina ua* [during] *diagnosis, e cramps legs. Na ave i a* [We took him to] [the neurologist] *e fai lana video* [to record] *I lana vae pe a migi foi gale* [his legs when cramped]*. E tusa e fai laga suesuega poo se* [It is about researching whether this is] *part you know, maybe that is the start of it* [Parkinson], *the muscles and the nerves’. (C5)*. These narratives illustrate how participants and caregivers navigated conflicting advice from traditional healers and medical professionals, contributing to uncertainty about the condition. It was also about the lack of understanding of what causes this disease and the unfamiliarity with the name and meaning of Parkinson’s, based on their own experiences and cultural narratives.

Pacific people are known for their strong spiritual beliefs. In this sense, one participant also mentioned how her faith supports her and described, *‘I’m always strong faith. That is my strength. That is my strength from God. That’s why I’m always put my heart myself to God’. (P9*). However, one patient noted that cultural beliefs contribute to stigma around Parkinson’s disease in the community, ‘*when my grandfather had it, it wasn’t spoken freely amongst the Polynesians, even though I should be only talking about my family, it’s kind of an old school sort of fear, and they don’t really respond openly, but maybe we were a lot better’. (P11).*

#### 3.1.4. Systematic Challenges

Systematic struggles address limited awareness and unmet needs (gaps in understanding and support). There was unfamiliarity with the illness and with the support available to both patients and caregivers. Caregivers bear a significant responsibility for caring for their loved ones while also managing their own well‐being and their families’ needs. During Talanoa sessions, it became apparent that many caregivers and patients lacked knowledge about PINK1 Parkinson’s disease. Some described it as a newly encountered illness, expressing unfamiliarity with its symptoms and with whether a cure is available. For example, a patient shared, *‘Confused I don’t know how I got it. I started talking about it with Dad and asking Dad if anyone from his past has got it or my mum and her side and none has got it’ (P4).* Similarly, her caregiver mentioned, *‘Yea ahh I was confused I don’t know where that sort of sickness comes from because on my side none of my parents or grandparents none of them, they got the Parkinson’. (C2).* Boosting awareness and knowledge of the disease can help patients, as one patient expressed relief upon understanding that the disease has a genetic origin*: ‘I felt a lot of relief because it actually gives you a reason how I got it through genetics. And yeah, it gave it an origin, which was like, great’ (P11).* One participant directly mentioned PINK1 Parkinson’s disease and stated, *‘I feel like there isn’t enough information on PINK1 that can be absorbed by people my age. Yeah. I guess it’s not simplified enough for us to understand what it is’. (P10).* Similarly, a lack of awareness of available support services and how to access assistance throughout their journey can be frustrating. As one participant explained, *‘But I don’t know what support should be available to be able to demand it, because I would demand it, but I don’t know what it should be. And there isn’t anyone we’ve reached out to the Parkinson society in …. But again, he’s like, the only person with this type of (Parkinson’s) like as nice as they’ve been, they don’t seem to know’. (C3).*

In some instances, there were also delayed or unmet patient needs. Reflecting on their experience, one caregiver described, *‘We need to get our bathroom sorted because it needs to be a little bit remodeled so that he can have an easier time getting in the shower. But I swear, it’s been like a year since we filled a paperwork out. We haven’t heard anything. You know what I mean?’ (C3*). The caregiver also mentioned a lack of professional staff (e.g., bicultural workers or nurses) and stated, *‘there should be a person that you can connect to that know how to help. And maybe it’s I don’t know, maybe because we don’t fit the income bracket of if we were, like, super-rich… And we don’t know how to navigate’. (C3*). Additionally, participants and caregivers reported receiving insufficient explanations about the side effects of prescribed medications and whether these treatments were effective. For example, one participant shared, *‘We are not totally sure yet in what medication regime works best or what dosage and stuff. We are not really we are still working that out’. (C3*).

Moreover, one caregiver highlighted disappointment with the support services provided for her husband’s mental health. While assistance was initially offered, the lack of follow‐up and ongoing support for PINK1 Parkinson’s disease, a lifelong condition, exacerbated the challenges faced by these families. The absence of sustained care has intensified the difficulties associated with managing the illness. They described *‘We got some support for that. But then when I don’t know I feel like you get the support and then it just falls away…He got some physiotherapy at first, and then they’re like, that was it. And I was like, but he should have it … because his disease is not changing. Kind of problem is I don’t know the New Zealand Healthcare system’. (C3*). Likewise, one patient, who lives alone, mentioned that mental health services were not fully supportive and shared *‘They only come and looking…ask me some question. They asked me about my Parkinson’s, my shaking. They thinking, uh, they can’t help it’. (P9).* In summary, the participants highlighted uncertainty about the illness’s origins and nature, as well as a desire for greater clarity, more information and better services.

In conclusion, Adaptation Struggles reflected the multidimensional challenges of Parkinson’s disease, requiring navigation of physical, emotional, social, cultural and systemic dimensions that disrupted participants’ daily lives and valued roles. This multidimensional nature of participants’ adaptation is associated with Pacific models of health, which conceptualise well‐being as encompassing interrelated physical, social, cultural, spiritual and family dimensions rather than focussing exclusively on disease symptoms [24, 25].

### 3.2. Theme of Positive Adaptation

Participants demonstrated Positive Adaptation to Parkinson’s disease through processes of normalisation and reliance on support networks.

#### 3.2.1. Normalisation

The participants living with PINK1 Parkinson’s disease collectively voiced a shared perspective on life, emphasising the importance of maintaining a normal lifestyle despite their challenges and framing their condition as just part of life rather than a disruption. They make an effort to maintain their daily routines and remain independent. Several participants echoed this sense of normalisation, for instance: *‘I can’t sleep during the daytime you know I wake up I go to the pub and play the machine. I work like a healthy man… So I just keep normal’. (P2)*. One participant, who had early‐onset PINK1 Parkinson’s disease like her mother, mentioned *‘I’m trying to stay as normal to what I was doing before, but now that I’m on medication, I’m actually able to freely do that’. (P10).* Another patient mentioned that *‘I’m not the only one that is going through tough times. We all are. So I’m pretty cool about that. It is what it is really’. (P11).*

Participants exhibited a strong sense of independence, consistently avoiding reliance on others, a theme prominently discussed across all Talanoa sessions. This independence reflects their determination to perform everyday tasks and activities as though unaffected by illness. Their unwavering fighting spirit and determination serve as a source of mental stability and provide a sense of normalcy in their lives. One participant described their independence: ‘*Because I can’t stay like Parkinson people and wait for the people feeding me. No, no, no I can make my food. I can do everything in my house washing, vacuum, drier’. (P2)*. Another patient shared, *‘I don’t feel society owes me anything. I don’t feel I should wave a flag and say, stop, stop, help me out. I’ve realised that I’m not bitter again. It’s like you got to pick yourself up’. (P11).*

#### 3.2.2. Support Networks

The support provided by families, friends and church communities plays a crucial role in assisting individuals living with PINK1 Parkinson’s disease. A key positive aspect shared by all participants was the central role of family as their primary support system. Participants expressed deep emotions when discussing the significance of their families in their health journeys. These experiences have not only strengthened family bonds but have also uplifted participants, highlighting the invaluable support provided by their family networks. For instance, one participant described: ‘*I am very thankful for the people around me and close to me they understand my situation’. (P1)*. Another participant also shared, *‘being around people really helped me because when I was diagnosed, it felt like the whole world just leaves you behind and you are sort of struggling quite a bit’. (P11)*.

The following quotations serve as a testament to the significance of a strong support system for individuals navigating PINK1 Parkinson’s disease. As one participant explained, *‘My church people they are all aware of my condition that is why of how grateful I am. You know around the community those people who knows me they are, so they support’. (P1)*. Some caregivers also expressed satisfaction with the support systems. As one caregiver stated, *‘We joined a Samoan group and yea a Parkinson group…They talk to each other. We do the exercise and all those stuff and it’s really good…You know have something that they can do every day instead of stay home and think about their sickness’. (C2).*

In summary, the ways that participants expressed their adaptation to the disease included maintaining family roles, seeking support from extended family or drawing on faith. These concepts capture how management of chronic illness among Pacific peoples is impacted by family, culture, spirituality and communication [26].

## 4. Discussion

This study explored the experiences of Pacific patients with PINK1 Parkinson’s disease and their caregivers. The findings are consistent with previous studies and highlight the roles of *Adaptation Struggles* (personal, social, cultural and systemic) and *Positive Adaptation* (normalisation of the illness and support networks).

Our participants reported some *Adaptation Struggles* that are similar to those seen in idiopathic Parkinson’s disease populations, such as challenges in maintaining social functioning and adapting to the progressive nature of Parkinson’s disease [13, 27–29]. However, consistent with prior research on Pacific people’s experiences with long‐term conditions [26], disruptions caused by motor and nonmotor symptoms were possibly experienced not merely as physical limitations but as a loss of contribution and connectedness within the family and community. Such changes led to social withdrawal or diminished participation in employment, reflecting the wider impact of illness on collective well‐being.

One aspect of Positive Adaptation, normalisation, describes the ongoing process by which people with Parkinson’s attempt to preserve normal routines, self‐identity and social roles, even as symptoms and limitations increase [30]. For many participants, this represented not only a coping mechanism but also a reflection of Pacific cultural values, in which health is perceived as functional—defined by the ability to perform daily tasks and fulfil social roles [26]. Furthermore, the emergence of support networks as a key theme in this study reinforces existing evidence highlighting their crucial role in the lives of people with Parkinson’s disease. Consistent with prior research, social support enables individuals with Parkinson’s disease to sustain their perceived physical health, emotional well‐being, social inclusion and engagement in daily and occupational roles [30]. Importantly, such networks not only foster a sense of connectedness and continuity for patients but also enhance caregivers’ resilience and reduce their psychological distress [31]. Within the Pacific context, these findings may be further interpreted through the cultural emphasis on collective well‐being and interdependence, where family and community networks are central to coping and adaptation [26].

Spiritual and cultural beliefs, including acceptance and fatalism, also shaped responses to illness—sometimes providing comfort, but at other times highlighting passive coping and delayed help‐seeking. Stigma arose from misconceptions, cultural beliefs and limited public awareness, which could negatively affect mental health, contributing to shame, social withdrawal, anxiety, depression and reduced participation in work and social life [13, 27]. As found in prior research, disease‐related knowledge among patients and caregivers is essential for effective health management and medication adherence [32, 33]. However, in the current findings, adaptation to Parkinson’s was further complicated by limited disease awareness, difficulties understanding health information and socioeconomic constraints, which have been similarly identified across studies of Pacific people with long‐term conditions in New Zealand [26]. These findings suggest that adaptation and social functioning among Pacific people with PINK1 Parkinson’s disease are influenced by an interplay of cultural, relational and systemic factors, highlighting the need for holistic, family‐centred and culturally responsive models of care [26, 34]. However, ‘Pacific people’ is not a single or unique ethnicity, and there are extensive differences between ethnic groups in country of birth (overseas‐born vs. NZ‐born) and in patterns of ethnic identification [35]. Spirituality is a central aspect of many Pacific worldviews, which is closely entangled with cultural identity, family, ancestors and interactions with the natural environment [36]. Pacific spirituality has roots in Indigenous belief systems for many communities, and Christianity has become integrated with these traditions as a layer rather than replacing them [36]. Consequently, Pacific worldviews are diverse and should not be taken as echoing a single, standardised set of practices or beliefs.

The findings indicate that support networks and normalisation are central to the lived experience of Pacific people with PINK1 Parkinson’s disease. Clinically, healthcare providers should incorporate family‐centred and culturally tailored approaches to care, recognising the importance of both social and spiritual support in promoting adaptation. One important consideration is the inclusion of bicultural health workers who can bridge the community and the health system. This approach is supported by Bramble et al. [37]; who found that assigning specialist Parkinson’s nurses in primary care settings helped prevent avoidable hospital admissions, reduce pressure on families and improve the quality of life for people living with Parkinson’s. Health services may also consider developing educational programmes to enhance patients’ and caregivers’ disease‐related knowledge, thereby supporting medication adherence and self‐management. In line with the need for tailored approaches to reduce health inequities among Pacific peoples [18], culturally tailored education and community engagement can enhance Pacific peoples’ awareness of PINK1 Parkinson’s disease, support early diagnosis, strengthen support networks and facilitate patients’ adaptation to and normalisation of their condition.

Unlike idiopathic Parkinson’s, people with PINK1 EOPD can get a definitive diagnosis through genetic testing. Therefore, an important aspect of managing PINK1 Parkinson’s disease is early diagnosis, which enables individuals to establish effective daily routines, adapt to living a normal life and build supportive networks, such as peer support groups. One study reported that, compared with those with later‐onset Parkinson’s disease, people with young‐onset Parkinson’s disease generally maintained a better quality of life and slower disease progression over time, although they experienced greater financial challenges because the disease occurred during their working years [38]. One explanation is that younger people may adapt or normalise living with Parkinson’s disease earlier in the disease course, in line with our findings. Within this context, another recent study found that young‐onset of Parkinson’s disease has profound psychosocial consequences, including stigma, shame, altered identity, disruption to employment and family roles, grief over anticipated losses and uncertainty about the future [39]. Therefore, early diagnosis also facilitates timely access to optimal medication regimens, culturally appropriate mental health support and tailored education that enhances patients’ and caregivers’ disease‐related knowledge and self‐management skills. Future efforts should focus on improving early diagnosis through genetic testing, developing culturally informed education and outreach initiatives and training primary care staff to recognise early symptoms and practise in a culturally responsive manner. Involving bicultural health workers can further strengthen trust and reduce diagnostic delays, leading to more timely interventions and improved outcomes.

Healthcare providers may adopt holistic, culturally responsive approaches informed by Pacific models of health, such as the Fonofale Model, by exploring the impact of Parkinson’s disease on physical, mental, spiritual, family and social well‐being and involving family in care planning where appropriate. To adopt such an approach, they need cultural competency (knowledge about cultures) and cultural safety (acknowledging one’s own power, attitudes, biases and assumptions), both of which are required to achieve Indigenous health [40]. Pacific cultural knowledge can help non‐Pacific allied health clinicians provide better culturally responsive healthcare [41]. A culturally responsive approach can include person‐centred care by acknowledging the diversity of Pacific peoples, involvement of family in care where suitable, exploring the effect of Parkinson’s disease on family and community roles, discussing spiritual support based on the individual’s preferences and working with Pacific health services or community organisations when further cultural support is required. In this context, general practitioners and practice nurses are well positioned to provide culturally tailored education on Parkinson’s disease, involve family members in discussions where appropriate and coordinate referrals to rehabilitation and community support services [42]. Moreover, Pacific health navigators can facilitate culturally responsive care by bridging communication and cultural gaps between healthcare providers and Pacific patients, supporting patient education, care coordination, advocacy and family engagement to improve access to healthcare [43].

Interventions for patients and caregivers may include culturally responsive health education programmes to improve self‐management skills and health outcomes for Pacific peoples, especially in partnership with, and preferably led by, Pacific peoples with lived experience and cultural knowledge to address the broader social, cultural and economic factors that influence health [44]. These programmes can use culturally relevant languages, customs and community leadership to deliver programmes in community or church settings, through group‐based, family‐centred and multidisciplinary approaches, by trained peer educators or community health workers, to improve engagement and self‐management [45]. Future research should investigate the effectiveness of such interventions in enhancing health outcomes and mitigating *Adaptation Struggles* within Pacific communities. Moreover, future research should explore Pacific peoples’ understandings and experiences of genetic information in the context of chronic and neurodegenerative conditions, including hereditary forms of Parkinson’s disease such as PINK1‐related disease. This is particularly important given the limited literature examining how cultural and familial perspectives shape interpretations of inheritance, genetic risk communication and decision‐making within Pacific families.

## Funding

This study was supported by R&AJ Francis Trust, 2106PGR. Open access publishing facilitated by The University of Auckland, as part of the Wiley ‐ The University of Auckland agreement via the Council of Australasian University Librarians.

## Conflicts of Interest

The authors declare no conflicts of interest.

## Data Availability

The data that support the findings of this study are available on request from the corresponding author. The data are not publicly available due to privacy or ethical restrictions.
